# Anti-obesity Medication (Naltrexon/ Bupropion) in Obesity Management: Single-Centre Early Experience

**DOI:** 10.21315/mjms-11-2024-879

**Published:** 2025-04-30

**Authors:** Siow Foon Tan, Firdaus Mukhtar, Ooi Chuan Ng, Barakatun Nisak Mohd Yusof, Nur Adilah Muhammadun Basar, Thanalactchumy Chandrabose, Cheng Xiang Jun, Zubaidah Nor Hanipah

**Affiliations:** 1 Pelabuhan Klang Primary Care Clinic, Pelabuhan Klang, Selangor, Malaysia; 2 Department of Psychiatry, Hospital Sultan Abdul Aziz Shah, Universiti Putra Malaysia Malaysia, Serdang, Selangor, Malaysia; 3 Department of Medicine, Hospital Sultan Abdul Aziz Shah, Universiti Putra Malaysia, Serdang, Selangor, Malaysia; 4 Department of Dietetics, Hospital Sultan Abdul Aziz Shah, Universiti Putra Malaysia, Serdang, Selangor, Malaysia; 5 Department of Rehabilitation, Hospital Sultan Abdul Aziz Shah, Universiti Putra Malaysia, Serdang, Selangor, Malaysia; 6 Department of Pharmacy, Hospital Sultan Abdul Aziz Shah, Universiti Putra Malaysia, Serdang, Selangor, Malaysia; 7 Department of Surgery, Hospital Sultan Abdul Aziz Shah, Universiti Putra Malaysia, Serdang, Selangor, Malaysia; 8 Faculty of Medicine and Health Sciences, Universiti Putra Malaysia, Serdang, Selangor, Malaysia

**Keywords:** naltrexone, bupropion, antiobesity medication, craving

## Abstract

**Background:**

Naltrexone/bupropion is a recently approved antiobesity medication in Malaysia. We aimed to determine the 12-week outcomes of naltrexone/bupropion as an antiobesity medication at our centre.

**Methods:**

This was a single-centre, prospective, pilot, observational study. All patients prescribed naltrexone/bupropion at Metabolic and Obesity Clinic, Hospital Sultan Abdul Aziz Shah, Universiti Putra Malaysia, between July 2023 and January 2024 were identified. The collected data included demographics, comorbidities, baseline weight, and body mass index. At the 12-week follow-up, data on weight, body mass index, side effects, and anti-craving effects were documented. Data are presented as mean ± standard deviation (SD) for continuous variables and as count and frequency for categorical variables.

**Results:**

Eighteen patients were treated with naltrexone/bupropion during the study. The majority were female (*n =* 13, 72%) and Malay (*n =* 13, 72%), with a mean age of 45 years (SD = 10). The mean body mass index was 38 kg/m^2^ (SD = 8). Comorbidities included diabetes (*n =* 9, 50%), hypertension (*n =* 7, 39%), and dyslipidaemia (*n =* 11, 61%). Of the 14 patients (78%) who completed 12 weeks of follow-up, the mean weight loss was 2.11 kg (SD = 2.61, *P =* 0.01), and the percentage of total body weight loss was 2%. The main side effects were giddiness (50%), nausea (44%), and headaches (38%). Naltrexone/bupropion was effective in reducing cravings in 75% of the patients. 63% of patients experienced side effects, which slowed escalation.

**Conclusion:**

Our early experience with naltrexone/bupropion as an antiobesity medication suggests that it is effective in managing obesity with craving symptoms. A longer follow-up with a larger group of patients is necessary before a definitive conclusion can be made.

## Introduction

Obesity is defined as excessive weight gain due to adipose tissue deposition in the body, which affects an individual’s biophysical and biopsychosocial aspects. The risk factors or comorbidities observed in the obese population include diabetes mellitus, cardiovascular disease, hyperlipidaemia, hypertension, and various cancers (1, 2). Owing to increased risk factors, the risk of mortality is high in obese individuals (3). Obesity has become a global pandemic over the last 50 years, and its prevalence in Malaysia has increased significantly (2). According to the National Health Morbidity Survey (NHMS) 2023 in Malaysia, the prevalence of overweight and obesity has increased by 6%, from 48% in 2015 to 54% in 2023 (4).

The Malaysian Clinical Practice Guidelines have clear guidelines for obesity classification based on body mass index (BMI) for Malaysian and Asian populations (5). This guideline aims to monitor the rising rates of obesity and identify at-risk individuals within the Malaysian population. The classification is as follows: overweight (BMI 23.0–27.4); obese class I (BMI 27.5–32.4); obese class II (BMI 32.5–37.4); and obese class III (BMI > 37.5).

Obesity management requires multiple approaches, including lifestyle, cognitive behavioural therapy, pharmacotherapy, and bariatric surgery (5). Research has reported that 5%–15% weight loss can significantly improve the metabolic risks involved, cardiovascular biomarkers, sleep cycle, and bone disorders, especially knee osteoarthritis (6). However, a qualitative study reported that such weight loss requires a certain degree of lifestyle modification, which is challenging (7). Of the above-mentioned approaches, pharmacotherapy is an adjunctive therapy that can act as a catalyst for the lifestyle management of obesity. Various antiobesity medications (AOM) have shown positive results in terms of weight loss and management (8). As per the latest data from the US Food and Drug Administration (FDA), six medications have been approved for obesity management, including orlistat, phentermine plus topiramate, phentermine, naltrexone and bupropion, liraglutide (3.0 mg/d), and semaglutide (2.4 mg/ wk) (5).

Naltrexone/bupropion (NB), extended-release and fixed-dose combination products were approved by the US FDA in September 2014 for use as antiobesity medications. Malaysia approved NB in June 2023 for use as an AOM. Naltrexone is an opioid antagonist, and the second molecule, bupropion, is an aminoketone antidepressant. Although the exact mechanism is unknown, preclinical studies have confirmed that both molecules work synergistically in the mesolimbic dopamine circuit and hypothalamus by enhancing satiety and reducing food intake, thus increasing energy expenditure (9). Bupropion stimulates hypothalamic pro-opiomelanocortin neurones, thus stimulating the release of α-melanocyte-stimulating hormone, exerting an anorectic effect, reducing food intake, and contributing to weight loss (10, 11). While bupropion alone produces modest weight loss (12), the effect is attenuated by β endorphin–mediated negative feedback. Naltrexone hooks this feedback, augments weight loss, and supports long-term weight maintenance (13). Working synergistically, they suppress appetite, control eating behaviour, and reduce food cravings, and studies have shown that they are effective in obesity management (13–15).

Although NB has been approved in Malaysia, local data regarding its effectiveness and adverse effects are lacking. The main objective of this study was to assess the 12-week effects of NB on obesity management, particularly weight loss and food craving reduction. The study also aimed to record and examine the potential side effects of the treatment for each participant throughout the study period.

## Methods

### Design and Participants Recruitment

This was a single-centre, prospective, pilot, observational study. All patients prescribed NB as an AOM at the Metabolic and Obesity Clinic (MOC), Hospital Sultan Abdul Aziz Shah, Universiti Putra Malaysia, from July 2023 to January 2024, were recruited.

The MOC is a specialised facility for managing obesity and metabolic disorders through a multidisciplinary team of experts, including bariatric surgeons, endocrinologists, registered dietitians, clinical psychologists, rehabilitation physicians, and pharmacists. All patients were counselled and followed by clinicians during the AOM administration. When NB was prescribed, patients were required to purchase it themselves as the medication was not approved for reimbursement or covered. Most AOMs were not subsidised for obesity treatment in Malaysia during the study period.

At MOC, patients undergo a thorough assessment during their initial visit, focusing on metabolic risk factors, obesity-related comorbidities, and mental health. Dietitians provide individualised dietary plans, and referrals to clinical psychologists are made when indicated. Additionally, patients were encouraged to achieve a minimum of 150 minutes per week of moderate-intensity exercise.

Pharmacists played an important role in this study by providing comprehensive counselling on the proper administration of NB, potential side effects, and the importance of adherence to the medication regimen. This ensured that patients were well informed and supported throughout their treatment, promoting better compliance and understanding of the medication.

The inclusion criteria for this study were as follows:

- A BMI ≥ 27.5 kg/m^2^ with weight-related comorbidities or BMI ≥ 30 kg/m^2^ with or without weight-related comorbidities;
- Prescription of NB as an AOM;
- The follow-up period was at least 3 months.

Patients were excluded for the following reasons:

- Did not fill a prescription because of high out-of-pocket costs, or
- They were on another AOM before their first MOC weight management programme appointment.

### Data Collection

Data were collected from electronic medical records, including demographics, comorbidities, baseline weight, and BMI. At week-12 follow-up, additional data were collected, including weight, BMI, NB side effects, anti-craving effects, and patients’ perception of NB.

### Outcome Measures

The primary outcome measure was the percentage of total body weight loss (TBWL) during the 12-week follow-up period. The secondary endpoints were the side effects of NB, its anti-craving effect, and the patient’s perception of the medication.

### Ethics Approval

Ethical approval for using human subjects was obtained from the Universiti Putra Malaysia (JKEUPM) (ethical approval number JKEUPM-2021-842).

### Statistical Analysis

Paired *t*-tests were used for continuous variables to evaluate the within-group differences. Normality tests for continuous variables were conducted using the Shapiro-Wilk test. Statistical significance was set at a two-tailed *P*-value of ≤ 0.05.

### Definition of Term Used

TBWL was defined as the absolute weight change (in kilograms) or as a percentage of weight loss from the initiation of AOM to the end of the intervention or follow-up period.

## Results

Eighteen patients were prescribed NB during the study period. The majority were females (*n* = 13, 72%) and Malays (*n* = 13, 72%), with a mean age of 45 years (SD = 10). The mean BMI was 38 kg/m^2^(SD = 8). Half (50%) of the patients were classified as obese (class III). Obesity-related comorbidities were dyslipidaemia (61.0%) and type 2 diabetes (50%), hypertension (39%), nonalcoholic fatty liver (28%) and obstructive sleep apnoea (11%).[Table t1-05mjms3202_oa]

The Shapiro-Wilk test was used to check the normality of continuous variables such as weight, systolic blood pressure (SBP), BMI, and diastolic blood pressure (DBP). These parameters were found to be normally distributed, with *P*-values greater than 0.05.

At the 12-week follow-up, 14 patients (78%) were still on NB. The proportion of mean weight loss was 2.11 kg (SD = 2.61), corresponding to 2% TBWL, which was significant (*P* = 0.01) (Table 2). There were no significant changes in systolic or diastolic blood pressure. The mean change in the blood pressures were 4.5 mmHg (SD = 8.3, *P* = 0.121, SBP) and 6.6 mmHg (SD = 12.5, *P* = 0.128, DBP).

**Figure 1:**
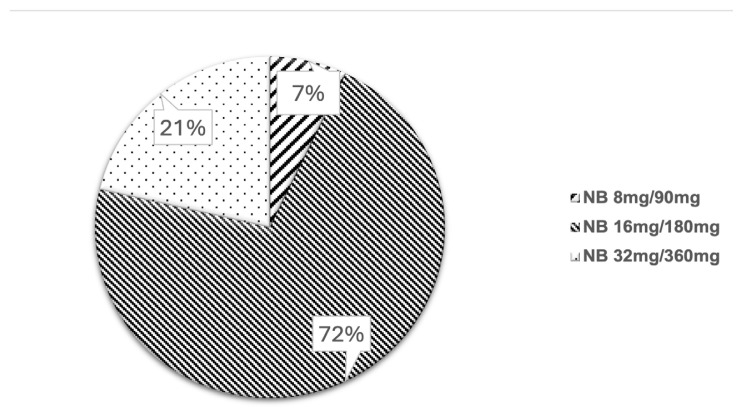
Percentage of patients on different doses of NB (*n* = 14)

Most (71%) were on half of the recommended therapeutic dosage of NB (naltrexone 16 mg/bupropion 180 mg). Only 21% of the patients were on the recommended therapeutic dosage of NB (Figure 1).

**Figure 2:**
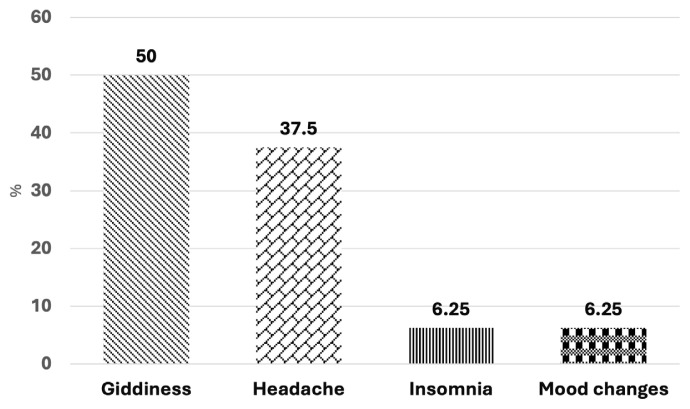
Percentage of central nervous system side effects with NB use (*n* = 16)

**Figure 3:**
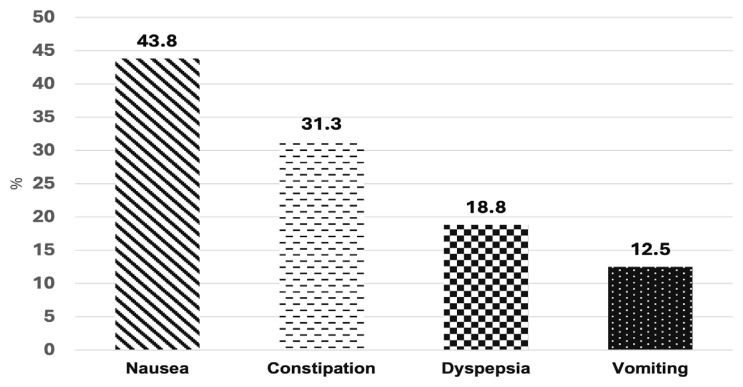
Percentage of gastrointestinal side effects with NB use (*n* = 16)

Most patients (75%) reported that NB effectively reduced cravings. 63% of the patients experienced side effects. The main gastrointestinal side effects were nausea (44%) and constipation (31%), and the central nervous side effects were giddiness (50%) and headaches (38%) (Figures 2 and 3). Two patients discontinued NB due to severe side effects, specifically headaches. Another two patients stopped NB because it was ineffective in reducing cravings (11%).

## Discussion

This is the first study to report on the early experience of using NB in Malaysia’s multidisciplinary weight management practice. We aimed to determine the 12-week outcomes of NB for managing obesity.

### Effect of NB on Weight Loss

Patients on NB for 12 weeks achieved a mean weight loss of 2.11 kg (SD = 2.61, *P* = 0.01), equivalent to 2% of their total body weight. This outcome is notably lower than the findings from a previous 16-week randomised controlled trial, which reported a 4% weight loss with naltrexone 50 mg/Bupropion 300 mg per day. (13) This outcome may be attributed to the suboptimal dosing used and compliance challenges commonly seen in real-world settings, both of which could impact the efficacy of NB. Most patients (72%) received a dose below the optimal level (naltrexone16 mg/bupropion180 mg per day), potentially limiting the full effect of NB on weight loss. The side effects and financial constraints likely contributed to the lower dose observed.

A longer follow-up (24 weeks) resulted in slightly higher weight loss (5%) (16). A similar dose of NB in a multidisciplinary weight management programme demonstrated better long-term effects, with 8% and 12% weight loss at 12 and 24 months, respectively (17). However, the sample size was small, and the retention rates declined.

### Effectiveness in Craving Management

Historically, bupropion and naltrexone have been used to treat nicotine and alcohol/opioid dependence, respectively. Simultaneously, these medications have shown effectiveness against food cravings and gain control over eating (16). Food cravings and emotional eating often hinder a reduced-calorie diet, making lifestyle modifications challenging and unsustainable (18). Our study confirmed that NB effectively manages obesity-related craving symptoms. A total of 75% of patients reported reduced cravings with NB. Subjective reports from patients who did not respond to NB (25%) could be due to several factors, such as emotional eating, binge eating, and low intrinsic motivation to lose weight, in which they claimed to expect the medication would instantly change their weight; hence, minimal effort has been made to change their behaviour or lifestyles. A study reported that more significant weight loss was achieved when NB was used as an adjunct to intensive behavioural modification (9%) (19).

### Side Effects of NB in Clinical Practice

This study assessed NB’s side effects of NBs in clinical practice. Overall, the frequency of side effects was lower than that reported in clinical trials (63% vs 83%) (15). Notably, our patients experienced higher rates of nausea (44% vs 30%), constipation (31% vs 16%), headache (37% vs 14%), and giddiness (50% vs 9%) and mood symptoms (6% vs 2%) (15). The insomnia rate was lower in our study than in the clinical trial (6% vs 8%) (15). None of the patients in this study reported suicidal ideation.

### Strengths, Limitations, and Recommendations of Future Studies

To the best of our knowledge, the current study is the first to evaluate the effectiveness of NB as an AOM in the Malaysian population. NB has demonstrated potential for managing obesity, notably by reducing cravings. Understanding craving symptom management and other side effects of medication highlights the importance of multidisciplinary teams in managing patients with obesity.

The current study has several limitations. The first limitation is the small sample size, which affects the generalisability of the results. The second limitation is the short follow-up period (12 weeks), considering that weight loss is a qualitative aspect and individual responses may vary; therefore, weight loss and long-term maintenance evaluation are problematic in this short period. To address these limitations, conducting studies with larger sample sizes and more extended follow-up periods is imperative further to evaluate NB’s safety, efficiency, and efficacy.

Suboptimal dosing and compliance issues may have affected the outcomes. Most patients receive doses below the optimal level, potentially limiting the effectiveness of the medication. Since patients had to purchase NB themselves, as AOM was not subsidised for obesity treatment during the study period, financial constraints may have influenced dose escalation. Patients might have opted for lower doses due to cost considerations despite clinical requirements. Future studies should consider strategies for improving adherence. Additionally, potential biases, such as information bias, measurement bias, and observer bias, due to using electronic medical records could have influenced the study findings. Standardised data collection procedures and training to obtain measurements are suggested to minimise these biases.

The influence of non-pharmacological components, such as diet, exercise, and cognitive behavioural therapy, on outcomes cannot be ruled out. Future studies should aim to isolate the effects of medication by controlling for these variables. Finally, the subjectivity in patient self-reports on cravings and side effects may introduce bias. Using objective measures and validated questionnaires to assess these outcomes is recommended in future research.

## Conclusion

Our findings highlight the effectiveness, safety, and practical considerations associated with NB as an AOM. In our initial observations, NB appeared to have potential benefits in managing obesity, especially in reducing cravings. However, further research with a larger and more diverse sample size is needed to confirm these findings.

## Figures and Tables

**Table 1 t1-05mjms3202_oa:** Baseline characteristics of participants (*n* = 18)

Characteristics	*n* (%), mean (SD)
Gender	
Male	5 (28)
Female	13 (72)
Age	45 (10)
Ethnicity	
Malay	13 (72)
Chinese	2 (11)
Indian	1 (6)
Others	2 (11)
Weight (kg)	98 (18)
BMI (kg/m^2^)	38 (18)
Obesity classification	
I (BMI 27.5–32.4 kg/m^2^)	5 (28)
II (BMI 32.5–37.4 kg/m^2^)	4 (22)
III (BMI>37.5 kg/m^2^)	9 (50)
Type 2 diabetes	
Yes	9 (50)
No	9 (50)
Hypertension	
Yes	7 (39)
No	11 (61)
Dyslipidaemia	
Yes	11 (61)
No	7 (39)
Obstructive sleep apnoea	
Yes	2 (11)
No	16 (89)
Fatty liver	
Yes	5 (28)
No	13 (72)

Notes: *n* = number; SD = standard deviation; BMI = body mass index

**Table 2:** Results of paired *t*-test for variables at baseline measurement to 12-week follow-up

Clinical parameters	Baseline (before on NB)	At 12 weeks follow-up^a^	Mean difference	*t*-value (df)	*P*-value
Weight (kg)	98.02 (18.24)	95.91 (17.89)	−2.11 (−2.61)	−3.03 (13)	0.010
BMI (kg/m^2^)	37.28 (6.65)	36.53(6.56)	−0.74 (−0.88)	−3.15 (13)	0.008
SBP (mmHg)	121.7 (9.79)	126.2 (7.32)	4.5 (8.3)	1.71 (9)	0.121
DBP (mmHg)	74.1 (7.56)	80.7 (11.45)	6.6 (12.5)	1.67 (9)	0.128

Notes:

a Mean (SD);

df = degree of freedom; NB = naltrexone/bupropion; SBP = systolic blood pressure; DBP = diastolic blood pressure; BMI=body mass index
